# Heat diffusion-related damping process in a highly precise coarse-grained model for nonlinear motion of SWCNT

**DOI:** 10.1038/s41598-020-79200-6

**Published:** 2021-01-12

**Authors:** Heeyuen Koh, Shohei Chiashi, Junichiro Shiomi, Shigeo Maruyama

**Affiliations:** 1 Mechanical and Aerospace Engineering Department, Seoul National University, 1 Gwanak-ro, Gwanak-gu, Seoul, 08826 South Korea; 2 Department of Mechanical Engineering, The University of Tokyo, 7-3-1 Hongo, Bunkyo-ku, Tokyo, 113-8656 Japan; 3 Energy Nano Engineering Lab., National Institute of Advanced Industrial Science and Technology (AIST), Ibaraki, 305-8564 Japan

**Keywords:** Materials science, Mathematics and computing, Nanoscience and technology, Physics

## Abstract

Second sound and heat diffusion in single-walled carbon nanotubes (SWCNT) are well-known phenomena which is related to the high thermal conductivity of this material. In this paper, we have shown that the heat diffusion along the tube axis affects the macroscopic motion of SWCNT and adapting this phenomena to coarse-grained (CG) model can improve the precision of the coarse-grained molecular dynamics (CGMD) exceptionally. The nonlinear macroscopic motion of SWCNT in the free thermal vibration condition in adiabatic environment is demonstrated in the most simplified version of CG modeling as maintaining finite temperature and total energy with suggested dissipation process derived from internal heat diffusion. The internal heat diffusion related to the cross correlated momentum from different potential energy functions is considered, and it can reproduce the nonlinear dynamic nature of SWCNTs without external thermostatting in CG model. Memory effect and thermostat with random noise distribution are not included, and the effect of heat diffusion on memory effect is quantified through Mori–Zwanzig formalism. This diffusion shows perfect syncronization of the motion between that of CGMD and MD simulation, which is started with initial conditions from the molecular dynamics (MD) simulation. The heat diffusion related to this process has shown the same dispersive characteristics to second wave in SWCNT. This replication with good precision indicates that the internal heat diffusion process is the essential cause of the nonlinearity of the tube. The nonlinear dynamic characteristics from the various scale of simple beads systems are examined with expanding its time step and node length.

## Introduction

Thermal energy is highly important for the dynamics of systems on the nm–$\upmu$m scale. Understanding how random fluctuation^1–5^ from thermal energy operates the dynamics in this range could expand the capability of the simulation model which should compromise the detailed dynamics from atomic scale phenomena^3,6,7^. The effort to parameterize and reveal the detailed mechanism for hierarchical structures^8–15^ often reaches continuum scale expression as an effective descriptor^16–20^. The validation of these trials has been demonstrated its capability to delineate the role of thermal motion in macroscale through the comparison of phonon dispersion relations^13,18,19^, which shows the coarse-grained description can manage thermal condition in atomic scale. The wide range of CG particles and time steps often leads to a memory effect in the equations of motion and behavior as a non-Markovian system^21^. The development of theoretical approaches to practically treat the memory effect and random noise distribution in more precise procedures for various simulation scales and models is a subject of ongoing research^21–23^.

Coarse-grained simulations of single-walled carbon nanotubes (SWCNTs) have been used to study the morphology of complex composites for theoretical and practical applications. Quantitative validation of coarse-grained (CG) modeling has been attempted using various methods, mostly for static characteristics^2,24–29^. The parameters for coarse graining SWCNTs have been fully explored for various SWCNT sizes^2,24,25^, and the dynamic characteristics in acoustic dissipation arising from global deformation have been investigated^24,30^. Moreover, CGMD simulations suggesting the structure of the SWCNT complex have been performed to determine the thermal conductivity and mechanical properties of composites, which are strongly dependent on the morphology characteristics of the composite^26,27,31^. For example, CGMD simulations were used by Won et al.^31^, for CG modeling of a vertically aligned SWCNT forest (VA-SWCNT) obtained by the top-down method. The use of the structure directly duplicated from scanning electron microscopy (SEM) images enabled successful simulation of the role of each structure type in the VA-SWCNT forest. Further research on multiscale modeling that showed good efficiency and precision for the VA-SWCNT forest^28,29^ proved that even the dynamic replication of VA-SWCNTs in chemical vapor deposition (CVD) and its further processing^32^ are feasible.

The CG modeling for saving the computational expenses of atomic simulation directly means that the trial should lose its detailed dynamic characteristics caused by such condition. Most progressed CGMD simulation for morphology, such as VA-SWCNT forest^28,29^ or buckypaper^26,27^, has a cylinder shaped CNT to keep realistic dynamic and structural characteristics with taking computational expanse. Some exceptions are dissipative particle dynamics (DPD) modeling with polymers^33–35^ and the mathematical random network of the sparse entanglement^36^. In other studies as well^13,37^, composing coarse grained structure to maintain the dynamic features of individual molecule at certain level is essential to enhance the methodology to analyze in multiscale systems^26,38,39^.

Recently, based on MD simulations, Koh et al.^40^ reported that the bending motion of SWCNTs under thermal equilibrium conditions exhibits nonlinear characteristics. The whirling motion of SWCNTs appears repeatedly in the course of conventional planar bending motion. This whirling motion also changes its rotational direction in each appearance. Successful CG modeling of SWCNTs should show the same motion characteristics as reported^40,41^. Any molecule which has one dimensional shape with fixed ends is suspected to have the similar nonlinear macroscopic motion characteristics according to the theoretical approach. The research scope of this paper is focused on a better algorithm for the simple beads model, which is the most simplified version of SWCNT. Clear understanding on the role of thermal randomness to macroscopic motion would reduce the complexity of dynamics into a simple beads system and it could be validated through CGMD simulation. The result of this trial would give the causality of nonlinear dynamic characteristics in SWCNT so that it could be manageable at some level of engineering.

**Figure 1:**
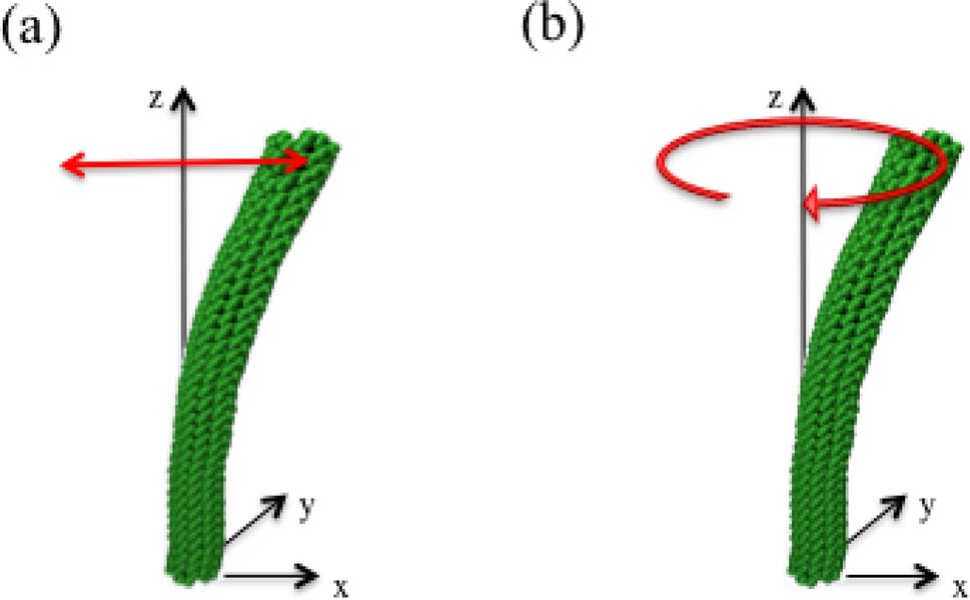
Visualization of motion from MD simulation. The average coordinate of each carbon ring perpendicular to tube axis is amplified by a factor of 5 to clearly visualize the motion: (**a**) planar bending motion, (**b**) nonplanar motion.

### Coarse-grained modeling for nonlinear motion in SWCNTs

The key feature of the nonlinear macroscopic motion in SWCNTs is the motion type exchange^40–42^. Each motion type as represented in Fig. 1 should appear repeatedly if the designed CG modeling has dynamic characteristics identical to those obtained in several atomic systems in simulations^40,41^ and experiments^43^. Judging by the high Q factor of SWCNTs, the use of quadratic functions to model the bond length and angle potential energy terms as harmonic potentials appears to be reasonable, but it does not ensure the nonlinearity of the motion type exchanges observed in the atomic-scale system since it is defined in a plane.

The governing equation for nonlinear bending^40^ describes the motion characteristics of a SWCNT that is coarse-grained along the tube axis and its analytic solution provided the nonlinearity including motion exchange, as shown in the MD simulation. For this nonlinear bending equation derived from Green–Lagrangian strain definition, the same strain definition can be applied to the coarse-grained model. According to the governing equation^40^, the nonlinear behavior results from the combination of the bending on two perpendicular planes and lengthwise deformation described by a single quadratic function. Thus, the Hamiltonian for SWCNTs that is identical to the Green-Lagrangian strain can be expressed as follows:

1 $$\begin{aligned} H_{MD}\left( x_1,\ldots ,x_n,p_1,\ldots ,p_n\right)= & {} \frac{1}{2} \frac{p^T p}{m} + \Phi _{MD}\left( x_1,\ldots ,x_n\right) , \end{aligned}$$

2 $$\begin{aligned} \frac{dx_i}{dt}= & {} \frac{\partial H}{\partial p_i}, \end{aligned}$$

3 $$\begin{aligned} \frac{dp_i}{dt}= & {} -\frac{\partial H}{\partial x_i}, \end{aligned}$$

4 $$\begin{aligned} \Phi _{MD}= & {} \Phi _{G.E.} + \Phi _{int}, \end{aligned}$$

5 $$\begin{aligned} \Phi _{G.E.}= & {} \frac{1}{2}k \left( w_l + w_{ \theta }\right) ^2, \end{aligned}$$

where $x_i$ and $p_i$ are the displacement and velocity of the coarse-grained particle, respectively. $w_l$ and $w_{ \theta }$ are the variables for bond length and angle deformation. The definition on these variables are in Supplementary Info. A. The angle definition in Supplementary Info A regards the SWCNT as an Euler beam. For the target system which is out of the limit, additional angle effect should be added^44^ to make it as Timoshenko beam model. $\Phi _{G.E.}$ is the potential energy given by the Green-Lagrangian strain definition, and $\Phi _{int}$ is the internal energy of the coarse-grained particle that is ignored in the governing equation. $\Phi _{G.E.}$ is defined as the quadratic function of the combination of bending, i.e., angle deformation and its deformation along the bond length. A full description of this expression is provided in Supplementary Info. A.

Ideally, a coarse-grained model should be composed of two independent Hamiltonian system for Stackel condition^45^, indicating that the momentum must be separated into two independent variables according to each potential energy type that is defined for each type of deformation.

6 $$\begin{aligned} H_{CG}= & {} H_{L} + {H}_{\theta }, \end{aligned}$$

7

8 $$\begin{aligned} {H}_{\theta }\left( \theta _1,\ldots ,\theta _n,p_{\theta ,1},\ldots ,p_{\theta ,n}\right)= & {} \frac{1}{2} \frac{p_{\theta }^{T} p_{\theta }}{m} + \Phi _{\theta } , \end{aligned}$$

where $H_L$ and $H_{\theta }$ are the Hamiltonians for the bond-length and angle deformations, respectively. $p_L$ and $p_{\theta }$ are the momenta for the two types of deformation.

However, in the case of coarse-grained molecular dynamics (CGMD) using a simple bead system, there is no momentum separation for each type of potential energy, which are defined separately so that there are only one type of momentum. We can separate the integrated value for each Hamiltonian:

9 $$\begin{aligned} p_{i}= & {} \left( p'^{\theta }_i+p'^{\ell }_i\right) \hat{e}_{p_i} \end{aligned}$$

10 $$\begin{aligned}= & {} p^{\theta }_i \hat{e}_{p_{\theta }}+p^{\ell }_i \hat{e}_{p_{\ell }}, \end{aligned}$$

where $p_{i}$ is the velocity of *i*th unit mass in CGMD. $p_i^{'\ell }$ and $p_i^{'\theta }$ are the momenta along the angle and bond length in the CGMD simulation, respectively. They are the scalar components of total velocity so that they share the unit vector $\hat{e}_{p_i }$, which is the direction of total velocity $p_i$. The components of $p_i$ from $p_i^{'\ell }$ and $p_i^{'\theta }$ are denoted as $p_i^{\ell }$ and $p_i^{\theta }$, with another unit vector set $\{\hat{e}_{p_{\ell }},\hat{e}_{p_{\theta }}\}$. The components of this set are the direction of each displacement variable, $\ell$ and $\theta$, respectively.

It is important to precisely define the direction of $p_i$ and $\dot{q}$, $\hat{e}_{\dot{q}_i}$. Due to the time integration and sharing of the momenta, the direction of the momentum of each component is designated at a certain moment through the bond length $\ell$ and angle $\theta$ as defined in Eq. (9), which are the variables of the potential functions at each atom. The unit vectors are the functions of the bond length $\ell$ and angle $\theta$, and decomposition of the momentum $p_i$ into $p_{i,\ell }$ and $p_{i,\theta }$ in orthogonal directions should be varied at every point in time.

## Results

### Influence of unseparated momenta

For a CG particle which is experiencing the shared momenta, as written in Eq. (9), the modified Hamilton’s principle becomes:

11 $$\begin{aligned} \delta I = \delta \int _{t_1}^{t_2}\left( \dot{q}_i p_i - H \left( q,\dot{q},t\right) \right) , \end{aligned}$$

with

12 $$\begin{aligned} H \left( q,\dot{q},t\right) = H_{\theta } + H_{\ell }. \end{aligned}$$

when we assume that $H_{\theta }$ and $H_{\ell }$ are two independent Hamiltonians, the least action principle should be valid for each with $\dot{\varvec{q}}$, which is the change in the displacement that a mass experienced in the phase space on both $H_{\theta }$ and $H_{\ell }$, simultaneously.

Using Eq. (9), it is possible to obtain the infinitesimal volume in the phase space for each type of Hamiltonian. We note that the momentum $p^{\ell }_i$ and $p^{\theta }_i$ are the function of displacement variables as shown in Eq. (9) so that the equations of motion are developed with additional damping term consequencially. For example, the volume of $H_{\ell }$ at a certain moment *t* is:

13 $$\begin{aligned} \dot{q} _i^{\ell }= & {} \frac{\partial H}{\partial p_i^{\ell }}, \end{aligned}$$

14 $$\begin{aligned} \dot{p}_i^{\ell }= & {} -\frac{\partial H}{\partial q_i^{\ell }} - \gamma p_i^{\ell } - \gamma ' \dot{q}_{i}^{\theta }, \end{aligned}$$

15 $$\begin{aligned} dq_i^{'\ell } dp_i^{'\ell }= & {} dq_i^{\ell } dp_i^{\ell } \left[ 1+\left( \gamma p_i^{\ell } + \gamma ' \dot{q}_{i}^{\theta }\right) \delta t \right] , \end{aligned}$$

where $\gamma$ and $\gamma '$ are for the proportionality of $\dot{q}^{\theta }_i$ and $p_i^{\ell }$ to $q_i^{\ell }$ in $H_{\ell }$ in Eq. (12) from the Taylor expansion, respectively. The differential is not vanishing due to the definition of unit vector in Eq. (10). We note that the damping in Eq. (15) has two different terms proportional to different types of momentum. The CGMD simulation with initial displacement and velocity from the MD simulation without extra thermostatting becomes the system calculated using the equation of motion in Eqs. (13)–(14). The result is given in Supplementary Info. A. Naturally, the simulation without external thermostatting would experience the damping in Eq. (14), and it is not adjusted to a specific temperature as given by the initial condition so that the temperature of CGMD simulation is in its equlibrium at approximately 1000 K, as shown in Supplementary Info. A. $\gamma$ and $\gamma '$ could be assumed to be time-varying variables, but we set their values to be constant in this study. Equation (15) makes the volume in phase space nonconservative with additional damping on $p_i$ and $q_i$.

### Memory effect from two independent Hamiltonians

To quantify the consequence of the unseparated momentum in Eq. (9) and its resultant, the damping in Eq. (14), the projection operator method used in the Mori–Zwanzig formalism is adapted from Kinjo and Hyodo^46^ and Kauzlaric^47^. In an atomic system that has a microscopic state *z* in the phase space $\hat{\Gamma }(t) = \{\hat{r}_{\alpha i},\hat{p}_{\alpha i}\}$ where $\hat{r}_{\alpha i}$ and $\hat{p}_{\alpha i}$ are the displacement, momentum and the Liouville operator, *L*, evolves with time^47^:

16 $$\begin{aligned} \dot{z}(t)= & {} L z, \end{aligned}$$

17 $$\begin{aligned} z= & {} exp(Lt) z_0. \end{aligned}$$

where $z_0$ is the initial state. The coarse-grained model that adapts center of mass (CoM) variables from *z* has the following notation:

18 $$\begin{aligned} \hat{R}_{\alpha }\equiv & {} \frac{\sum _i m_{\alpha i}\hat{r}_{\alpha i}}{M_{\alpha }}, \end{aligned}$$

19 $$\begin{aligned} \hat{P}_{\alpha }\equiv & {} \sum _i \hat{p}_{\alpha i}, \end{aligned}$$

20 $$\begin{aligned} M_{\alpha } \equiv \sum _i m_{\alpha i}, \end{aligned}$$

where $\alpha$ can be either $\ell$ or $\theta$ in the simple bead system. $m_{\alpha }$ is the mass of an atom. $\hat{R}_{\alpha }$. $\hat{P}_{\alpha }$ and $M_{\alpha }$ are the displacement, momentum and the inertia of CG particle, respectively. In the Mori–Zwanzig formalism^46–48^, the evolution of the variables for a coarse-grained particle as a function of a small, microscopic group, $A_{\mu }=A_{\mu }(z(t))$, is treated using the phase space density $f_s(\hat{\Gamma }_s(t),\hat{\Gamma }_s)$ for Hamiltonian *H* in the phase space coordinate of coarse grained system, $\hat{\Gamma }_s(t) \equiv \{\hat{R}_{\alpha }, \hat{P}_{\alpha }\}$^46^. $\hat{\Gamma }_s$ is the corresponding field variables^46^.

Dynamic variable $g(\hat{\Gamma }(t))$ can be defined on $f_s$ with the equilibrium distribution $\Psi (\hat{\Gamma })=e^{-\beta H}/Z$. The projection P for $g_P(\hat{\Gamma _s}(t))$ for and Q = 1-P can divide $g(\hat{\Gamma _s}(t))$ and $\left( \frac{d}{ds} \right) _{\Gamma } f_s$ as:

21 $$\begin{aligned} g\left( \hat{\Gamma _s}(t)\right)= & {} g_P\left( \hat{\Gamma _s}(t)\right) + g_Q\left( \hat{\Gamma _s}(t)\right) , \end{aligned}$$

22 $$\begin{aligned} \left( \frac{d}{ds} \right) _{\Gamma } f_s(\hat{\Gamma _s}(t);\Gamma _s)= & {} P iLf_s \left( \hat{\Gamma }_s(t);\Gamma _s\right) + Q iLf_s \left( \hat{\Gamma }_s(t);\Gamma _s \right) , \end{aligned}$$

where

23 $$\begin{aligned}{}&g_P\left( \hat{\Gamma }(t)\right) \equiv Pg\left( \hat{\Gamma }(t)\right) = \int d\Gamma '_s \int d\Gamma _s''f_s\left( \hat{\Gamma }_s\left( t_0\right) ;\Gamma _s'\right) \nonumber \\&\quad \times \left\langle f_s(\hat{\Gamma }_s(t_0);\Gamma _s')f_s(\hat{\Gamma }_s(t_0);\Gamma _s'') \right\rangle ^{-1}\nonumber \\&\quad \times \left\langle f_s(\hat{\Gamma }_s(t_0);\Gamma _s'') g(\hat{\Gamma }(t))\right\rangle \end{aligned}$$

24 $$\begin{aligned}&\left\langle f_s\left( \hat{\Gamma }_s(t_0);\Gamma _s\right) g_Q(\hat{\Gamma }(t)) \right\rangle =0, \end{aligned}$$

25 $$\begin{aligned}&\left( A,B \right) \equiv \left\langle A(\hat{\Gamma )},B(\hat{\Gamma }) \right\rangle = \int d\hat{\Gamma } A(\hat{\Gamma })B(\hat{\Gamma })\Psi (\hat{\Gamma }). \end{aligned}$$

Based on the assumption that the each Hamiltonian is independent, the integration of Eq. (23) is could be conducted on the phase space for either one of the Hamiltonian $H_{\ell }$ or $H_{\theta }$. The projector *P*, as well could be validated in the phase space either of $H_{\ell }$ or $H_{\theta }$. The phase space for $H_{\ell }$, for example, becomes:

26 $$\begin{aligned} f_{s\ell }\left( \hat{\Gamma }(t);\Gamma _s\right) \equiv \delta \left( \hat{\Gamma }_{s\ell }(t)-\Gamma _s\right) = \prod _{\ell } \delta \left( \hat{R}_{\ell }-R_{\ell }\right) \delta \left( \hat{P}_{\ell }-P_{\ell }\right) . \end{aligned}$$

Same condition in Eqs. (21)–(25) should be satisfied for $H_{\theta }$ with $f_{s\theta }$. $f_{s\theta }$ and $f_{s\ell }$ are the phase space density of the same atomic system but that of different expressions. Since the variables $\ell$ and $\theta$ are independent at each moment, the operator P on different phase space density $f_{s\theta }$ and $f_{s\ell }$ are as well orthogonal each other. Then, the time evolution of $f_{s\ell }$ is defined by its Hamiltonian with the momentum in Eq. (9) and Liouville theorem:

27 $$\begin{aligned} \left( \frac{d}{dt} \right) _{\Gamma } f_s= & {} -\sum _{\alpha } \sum _{i} \left\{ \frac{\partial H}{\partial \hat{r}_{\alpha i}} \cdot \frac{\partial }{\partial \hat{p}_{\alpha i}} - \frac{\partial H}{\partial \hat{p}_{\alpha i}} \cdot \frac{\partial }{\partial \hat{r}_{\alpha i}} \right\} f_s \nonumber \\= & {} - \sum _{\alpha } \left\{ \hat{F}_{\alpha } \cdot \frac{\partial }{\partial \hat{P}_{\alpha }} - \frac{\hat{P} _{\alpha }}{M_{\alpha }} \cdot \frac{\partial }{\partial \hat{R}_{\alpha }} \right\} f_s \equiv iL_s f_s, \end{aligned}$$

where

28 $$\begin{aligned} L_s\equiv & {} -\sum _{\alpha } \left\{ \hat{F}_{\alpha } \cdot \frac{\partial }{\partial \hat{P}_{\alpha }} - \frac{\hat{P} _{\alpha }}{M_{\alpha }} \cdot \frac{\partial }{\partial \hat{R}_{\alpha }} \right\} , \end{aligned}$$

29 $$\begin{aligned} \hat{F}_{\alpha }= & {} -\sum ^{n_{\alpha }}_{i} \frac{\partial U}{\partial \hat{r}_{\alpha i}} +\gamma \hat{P}_{\alpha }+\gamma ' \dot{q}_{\theta }, \end{aligned}$$

could be considered for each Hamiltonian. $L_s$ is Liouville operator and $\hat{F}$ is the force from the given Hamiltonian. $\hat{P}$ and $\hat{R}$ are the momentum and displacement of the CG particle as noted in Eqs. (18)–(20) The derivation of Eq. (29) has been dealt in the previous subsection for Hamilton’s least action. From the explicit definition of damping in Eq. (29), which is the sum of $\gamma \hat{P}_{\alpha }$ and $\gamma ' \dot{q}_{\theta }$, we are going to quantify the influence of this damping terms in time evolution. Let us suppose that we have only N CG particles in adiabatic condition which are obeying the Eqs. (27)–(29). This means that they are unaffected by other influences such as thermal fluctuations. $i L_s$ in Eq. (28) can be divided by operator *P*, with the additional damping $\gamma \hat{P}_{\alpha }\frac{\partial }{\partial \hat{P}}$ from the reference^46^, and *Q*, which now has a very specific definition, $\gamma ' \dot{q}_{\theta } \frac{\partial }{\partial \hat{P}}$. From the definition of *Q* that is defined explicitly under the assumption of adiabatic condition of the system, the terms for the fluctuation force and memory effect are changed so that the equation of motion from the time-evolution for the phase-space density of $f_{s\ell }$ becomes:

30 $$\begin{aligned} \frac{d}{dt}\hat{ P}_{\ell }=\frac{1}{\beta }\frac{\partial }{\partial \hat{R}_{\ell }} \ln \omega \left( \hat{ R}\right) - \gamma \hat{P}_{\ell }- \beta \sum _{N_\alpha }\int ^{t}_{0} ds<\left[ \delta P^{Q}_{\ell }(t-s)\right] \times \left[ \delta P^{ Q}_{\ell }(0) \right] ^{T} >\frac{\hat{P}_{\ell }(s)}{M_{\ell }} + \delta P^{Q}_{\ell }(t), \end{aligned}$$

where

31 $$\begin{aligned} P\left( \hat{\Gamma }(0),\Gamma _s\right)= & {} -\sum _{N_\ell } \left[ \gamma ' \dot{q}_{i,\theta }\cdot \frac{\partial }{\partial P_{\ell }} \left( \hat{\Gamma }_s-\Gamma _s\right) \right] , \end{aligned}$$

32 $$\begin{aligned} \int d \Gamma _{s} P_{\ell } P\left( \hat{\Gamma }(0),\Gamma _s\right)= & {} \delta P_{\ell }=\gamma ' \dot{q}_{\theta }, \end{aligned}$$

33 $$\begin{aligned} \delta P^{Q}_{\ell }(t)= & {} e^{QiLt} F\left( \hat{\Gamma }(0),\Gamma _s\right) . \end{aligned}$$

$N_\ell$ is the number of CG particle involved in the $H_{\ell }$. $P(\hat{\Gamma }(0),\Gamma _s)$ is originally noted^46^ as $F(\hat{\Gamma }(0),\Gamma _s)$ for the fluctuation force from the projection *Q* without explicit definition as we have shown in Eq. (29). Note that $\delta P^{Q}_{\ell }(t)$ is $\delta F^{Q}_{\sigma }(t)$ in the reference^46^. It is changed to reveal that the fluctuation arises from the momentum of the other Hamiltonian. Subscript $\ell$ can be changed to $\theta$ when the evolution on the $f_{s\theta }$ is considered.

More specifically, the memory effect integration, $M_{\ell }$ which is the second term in Eq. (32) becomes the following:

34 $$\begin{aligned}&- \beta \sum _{\alpha } \int ^{t}_{0} ds \left\langle \left[ \delta P^{ Q}_{\sigma }(t-s)\right] \times \left[ \delta P^{ Q}_{\alpha }(0) \right] ^{T} \right\rangle \cdot \frac{\hat{P}_{\alpha }(s)}{M_{\alpha }} \nonumber \\&\quad = - \beta \sum _{\alpha } \left\langle \left[ \int ^{t}_{0} ds \delta P^{ Q}_{\sigma }(t-s) \cdot \frac{\hat{P}_{\alpha }(s)}{M_{\alpha }} \right] \times \left[ \delta P^{ Q}_{\alpha }(0) \right] ^{T} \right\rangle , \end{aligned}$$

The value of time integration, $M_{\ell }$ in Eq. (34) has same value as the cross-correlation of these variables in the frequency domain:

35 $$\begin{aligned} M_{\ell }= & {} \int ^{t}_{0} ds \delta P^{ Q}_{\sigma }(t-s) \cdot \frac{\hat{P}_{\alpha }(s)}{M_{\alpha }}, \end{aligned}$$

36 $$\begin{aligned} M_{\ell }( \omega )= & {} \int dt e^{i\omega t} \int ^{t}_{0} ds \delta P^{ Q}_{\sigma }(t-s) \cdot \frac{\hat{P}_{\alpha }(s)}{M_{\alpha }} = \delta P^{ Q}_{\sigma }( \omega ) \hat{P}_{\alpha }( \omega ). \end{aligned}$$

**Figure 2:**
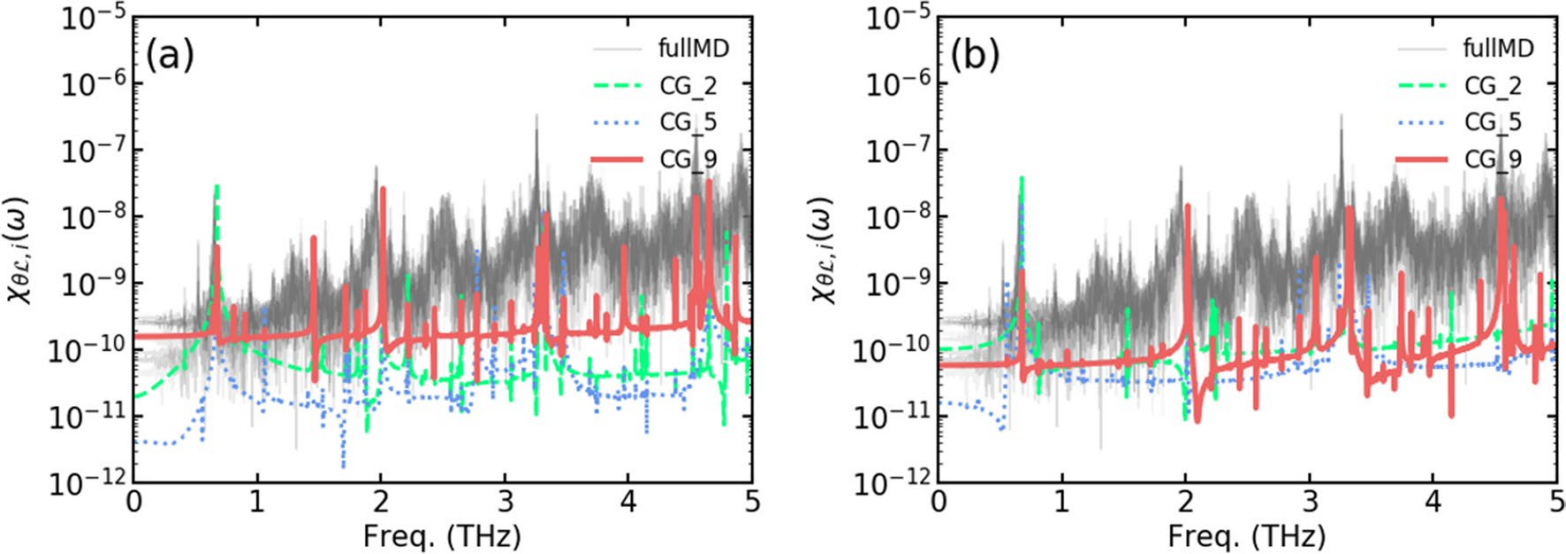
Cross-correlation from CGMD and the simple bead model from MD data in the frequency domain. The gray line indicates the MD simulation duplicated with all CG particles in the simple bead model; the green, blue and red lines are the CGMD results at the 2nd, 5th, and 9th nodes, respectively, obtained (**a**) without damping. The MD simulation results show a rather narrow range of distribution between each CG particle compared to the CGMD results. The peak of the CGMD results is close to the delta function, (**b**) with internal heat diffusion. The difference between each node in CGMD is narrowed and the peak at certain frequency is diminished compared to (**a**). The peaks are broadened.

$M_{\theta }(\omega )$ will share the same definition and the value theoretically with $M_{\ell }(\omega )$. The numerical calculation can distort the distribution of cross correlation from the $M_{\ell }$ slightly according to the finite length of the data and initial condition which are involved in calculation. Cross-correlation in the frequency domain is achieved by processing the CGMD and MD simulation data. The MD and CGMD simulations are carried out for (5,5) 8-nm-long SWCNTs, and the cross-correlation calculation is described in the Method section. This quantity of $M_{\theta or \ell }$ in CGMD simulation in Fig. 2a has a different peak shape from that of the MD simulation whose results are averaged as the simple bead system. No significant results have been found in the range below THz for both cases. The CGMD simulation results show more distinctive peaks than the MD simulation results. This observation means that (1) in the case of MD simulations, for the simple bead system with the displacement and velocity averaged from the MD results, the influence of the thermal fluctuation or heat bath should be incorporated as another dissipation term, which is absent in the definition of CGMD and which makes the momentum in two different Hamiltonian independent to each other, and (2) MD simulations have a cross-correlation that is not active in the bending frequency range but rather is active in the optical mode range. For the integrity of the paper, autocorrelation of the momenta in each Hamiltonian is presented in Supplementary Info. B, and it shows non-Markovian characteristics.

### Heat diffusion in the general Langevin equation

It is interesting to consider how the thermal random motion due to the heat bath can help to obtain a high Q factor of macroscopic motion through establishing two independent potential energy surface in equilibrium with the correct level of the cross-correlated state. All these complex situations are well summarized by the definition of free energy and irreducible friction for reversible state^49,50^. However, it is difficult to define the value at each time step while the simulation is on running.

The effect of the heat bath suggested by Zwanzig in 1961^51^ and described by an additional Hamiltonian $H_b$ represents the general influence of the collective dynamics from the Hamiltonian system belonging to the individual atoms inside the CG particle. From the THz range of cross correlated state in the previous section, we can assume that $2p^{'\ell } p^{'\theta }$ from the quadratic form of Eq. (9) is correspondingly from the thermal energy of the heat bath.

**Figure 3:**
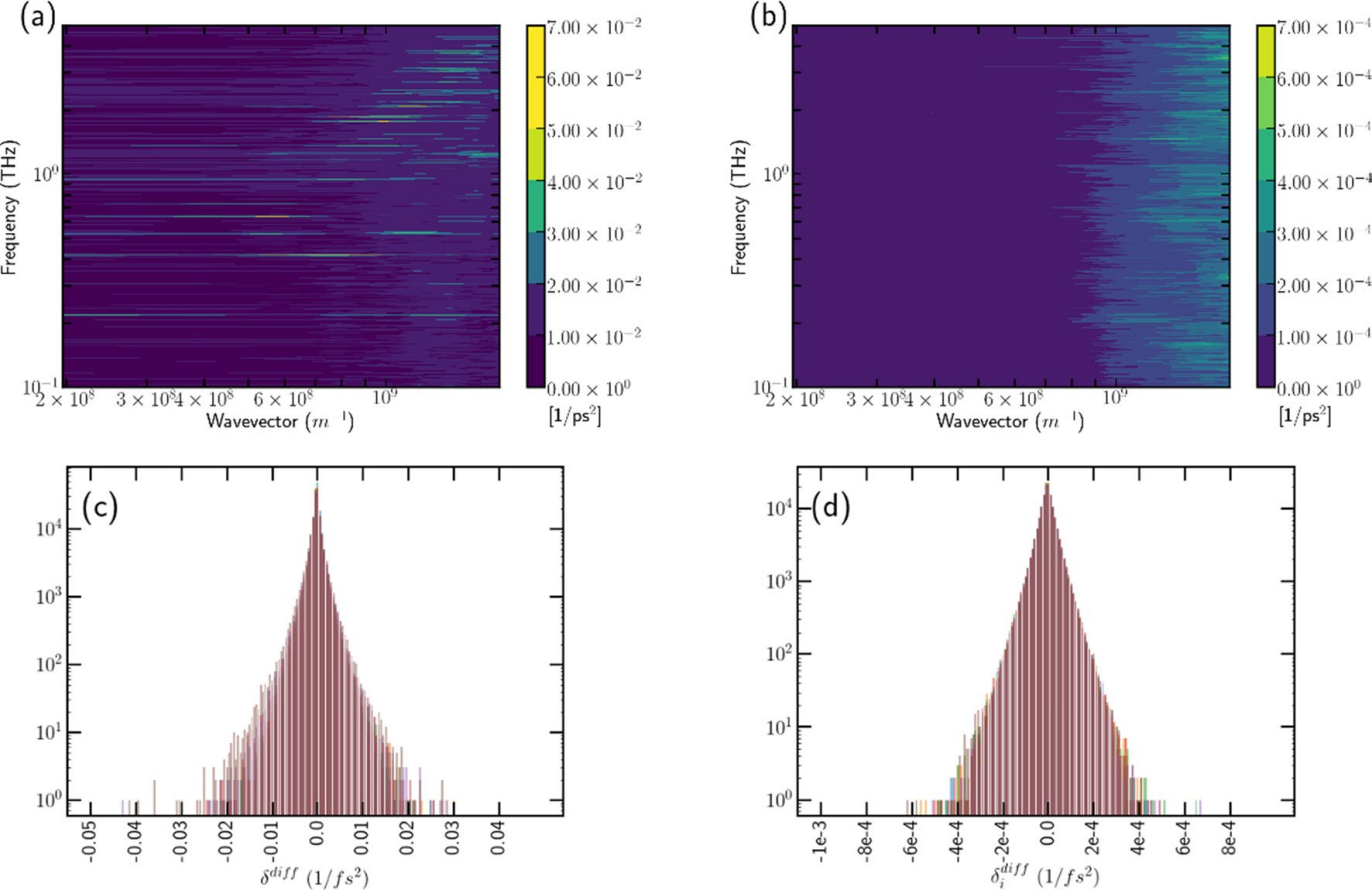
Contour of the value of $\delta ^{diff}$ along the tube axis. Each value is distributed with dt=10 fs along the *x* axis. (**a**) MD simulation result, (**b**) CGMD simulation results, (**c**) histogram of the diffused level in simple bead system from MD simulation result during 10 ns, with the results from all UAs shown, (**d**) histogram for the CGMD simulation results.

It could be presumed that a small amount of the disturbance in the kinetic energy of the CG particle such as $\delta KE$ in THz range which manipulates the macroscopic motion, as being involved in total kinetic energy of CG particle, $K_{tot}=KE_0+\delta KE$. If thermal energy from heat bath is affecting the macrosopic motion, phonon modulation at THz could be the reason, whose dynamics is ruled by the second sound which explains the momentum and energy balance of phonons^52^. Second sound modulation, $\partial ^2 u / \partial t^2 = \partial ^2 u / \partial z^2$, where *z* is the variable for the nanotube length axis can be considered, with the temperature deviation *u* which is caused by $p_{i}^{'l} p_{i}{'\theta }$ from the quadratic form of Eq. (9) and its diffusion should shown as the collective dynamics from the atomic simulation if the second sound is involved. It is a valid observable in the simplified beads model from MD simulation. The value of $\frac{\partial ^2 p_{i}^{'l} p_{i}{'\theta }}{\partial x ^2}$ in Fig. 3a shows dispersive characteristics as explained by Lee and Lindsay in 2017^52^. We can confirm that the amount of energy $\frac{\partial ^2 p_{i}^{'l} p_{i}{'\theta }}{\partial x ^2}$ is diffused as the second sound modulation in atomic simulation.

Instead of building the precise second sound modulation phenomenon in CGMD simulation, we can conjecture that the diffusion depends on the a set of macroscopic momentum $p^{'\ell }, p^{'\theta }$ in the way the function of the momentum can affect the coarse-grained system in a similar way of second sound modulation. Based on the direct proportionality between *KE* and *T*, the amount of cross-correlated state equivalent to $\frac{\partial ^2 p_{i}^{'l} p_{i}{'\theta }}{\partial x ^2}$ can be incorporated into the equations of motion for CG particle using the definition of the diffusion equation.The system with this diffusion process can be described as follows:

37 $$\begin{aligned} H_{CG}= & {} H_{s}(X)+H_{b}(X,Y), \end{aligned}$$

38 $$\begin{aligned} H\left( x_1,\ldots ,x_n,p_1,\ldots ,p_n\right)= & {} \frac{1}{2} \Sigma \frac{p_i^2}{m} + \Phi _{CG}(x_1,\ldots ,x_n), \end{aligned}$$

39 $$\begin{aligned} H_b \left( x_1,\ldots ,x_n,p_1,\ldots ,p_n\right)= & {} \delta KE = \Sigma _i \left\langle \delta _i^{diff} \right\rangle _{op}, \end{aligned}$$

40 $$\begin{aligned} \delta _i^{diff}= & {} \frac{ \partial ^2 p_{i}^{'l} p_{i}{'\theta }}{\partial x ^2}. \end{aligned}$$

This cross-correlation can be incorporated as a meta-dynamics derivation^53^ into the governing equation as defined in Eqs. (37)–(40), which is activated locally as a constrained term of the Lagrangian multiplier. Further approximation and derivation are provided in Method section and Supplementary Info. C. The equations of motion from this rough approximation is:

41 $$\begin{aligned} m\ddot{l}+\alpha \left\langle \frac{\partial ^2 v_{\theta } }{\partial x^2} \right\rangle _{op}= & {} - \frac{\partial \Phi _{CG}}{\partial l}, \end{aligned}$$

42where to give this virtual force in the THz range, the $+/-$ sign alternation is included, which is noted as $\left\langle \right\rangle _{op}$ in Eqs. (41)–(42). $\alpha$ and $\alpha '$ are the parameters for heat diffusion damping which are manually optimized. We suppose that these variables are related to the quantity of heat capacity of the group of atoms which is varying with the macroscopic deformation. More rigorous study would be interesting for further works on the connection of the diffusion of the internal heat affected by macroscopic deformation and how second sound modulation is involved.

In practical application of the damping from the heat diffusion, the optical mode definition of Eqs. (41)–(42) can bound the memory effect term $M(\omega )$ in the optical mode frequency range as well:

43 $$\begin{aligned} M= & {} \sum _{\alpha }\int ^{t}_{0} ds \delta P^{ Q}_{\sigma }(t-s) \cdot \frac{\hat{P}_{\alpha }(s)}{M_{\alpha }} \end{aligned}$$

44 $$\begin{aligned}= & {} \int ^{t}_{0} ds \dot{q}_{i}(t-s)e^{i \omega _{op} (t-s)} \cdot \frac{\hat{P}_{\alpha }(s)}{M_{\alpha }}\approx C e^{i \omega _{op} (t)}, \end{aligned}$$

where *C* is an arbitrary value. The time integration of the optical mode can be a damping on the infinitesimal volume in phase space as written in Eq. (15) so that infinitesimal volume can be preserved with small oscillation.

In Fig. 3b, the dispersion plot of $\delta _i^{diff}$ from CGMD calculated using Eqs. (41)–(42) shows no dispersion at all, which is quite natural since the equation of motion artificially provides perturbation in the THz range. However, the histograms of the value of diffusion in CGMD have identical distribution to that of the MD simulation, as shown in Fig. 3c,d. This result reflects the similarity of dynamics between CGMD and MD.

### Validation

**Figure 4:**
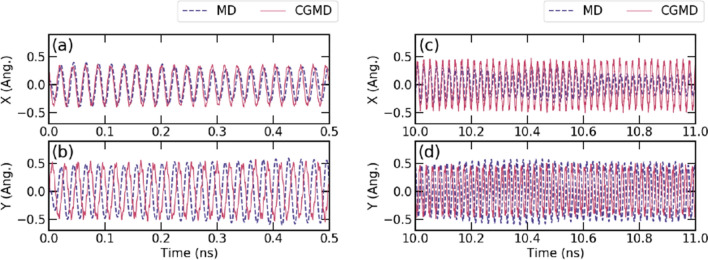
Initial trajectory of the SWCNTs calculated from MD and strain CGMD. The blue line shows the MD simulation results, and the red line shows the strain CGMD results. Strain CGMD obtains its initial data from the MD simulation, and there is no further compensation during the calculation process: (**a**), (**b**) displacements of the SWCNT tip along the *x* and *y* axes for the initial 0.5 ns, (**c**) and (**d**) displacements along *x* and *y* for 1 ns after 10 ns.

Figure 4 shows a comparison of the trajectories of the end of the tube from CGMD and from MD simulation whose simulation condition is described in Method section and Supplementary Info. D. The same timeline is displayed in the animated gif. The displacements of both conditions are processed by inverse fast Fourier transform (IFFT) to remove the higher frequency component. Without any additional data processing, the two simulations that share the initial conditions only give almost perfect synchronization from few Angstroms of its initial stage up to 10 ns. The result of the simulations with longer duration is validated by counting the number of motion exchange. The detailed result is also provided in Supplementary Info. D. It is concluded that the simulation with a soft boundary with 1 eV fixing with the Lennard-Jones (LJ) potential energy at the end of cantilever beam is the closest to the MD simulation result.

**Figure 5:**
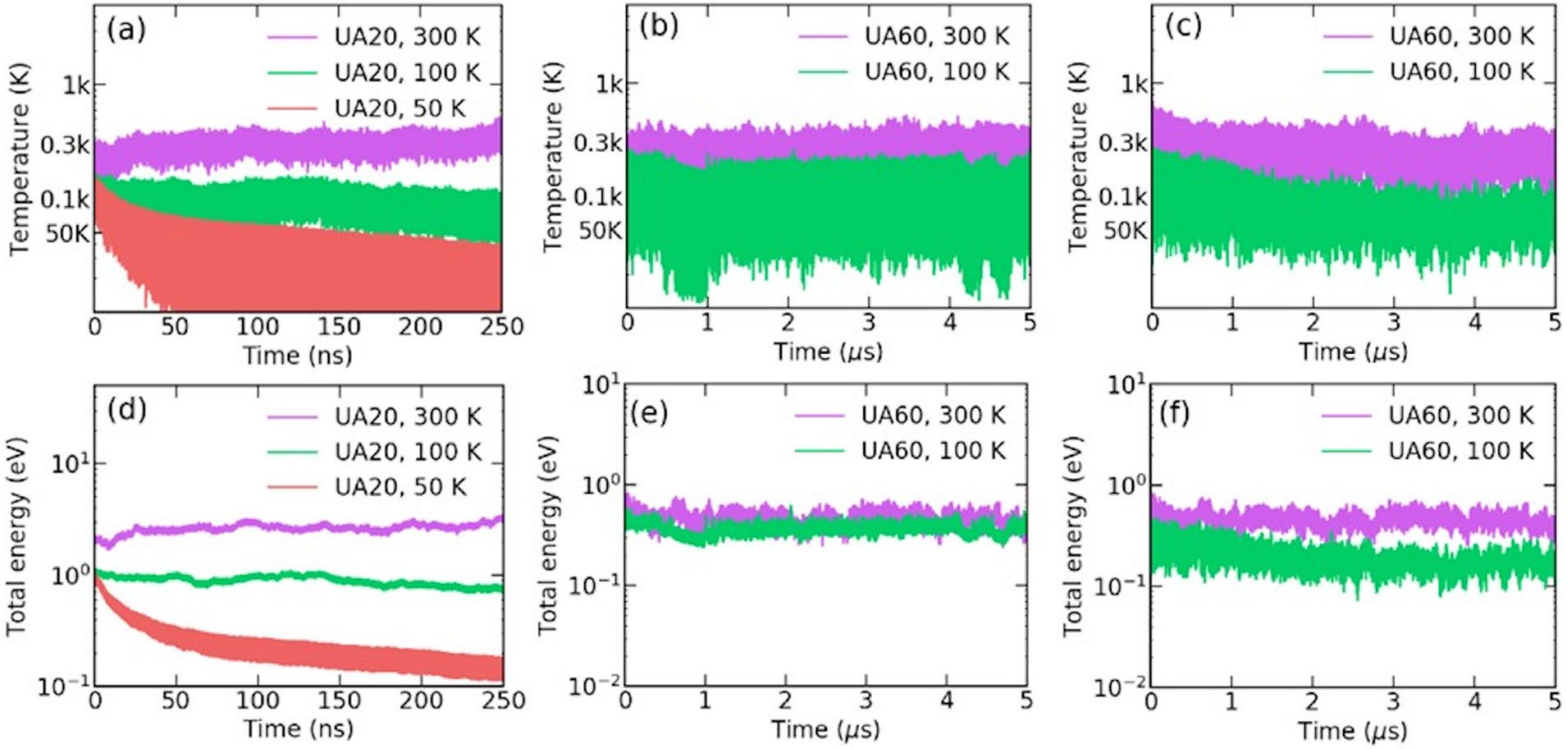
Temperature and total energy of the strain CGMD simulation without external thermostatting at 50, 100 and 300 K shown by red, green and magenta lines, respectively: (**a**) and (**d**) UA20 with rigid boundary, (**b**) and (**e**) UA60 with a 0 K rigid boundary, (**c**) and (**f**) UA60 with LJ potential fixation with $\varepsilon = 1$ eV for 300 K and 1 eV for 100 K.

The CGMD simulation calculated using the initial data of the MD simulation resolves the thermal equilibrium conditions well, so several simulations with different size of beads retains in constant temperature conditions as the given initial conditions. Total 3 different types of CG particle are conducted with 20, 60 and 120 carbon atoms of SWCNT. These are noted as UA20, UA60 and UA120, respectively. The results are as shown in Fig. 5. Figure 5a shows the results for the UA20 simulation with a rigid boundary. In case of 50 K, the right parameter set could not found so that it is not close the equilibrium. Figure 5b shows the results of the UA60 simulation, which also has a rigid boundary. Both results show the stabilized temperature level around 300 K, but the fluctuation is rather large in the case of UA60. Figure 5c with the LJ potential function for fixation with a condition similar to that the MD simulation shows less fluctuation. Figure 5d–f show the total energy for each case, which remains at a constant value with a given random force in Eqs. (41)–(42). We do not argue further regarding whether the suggested calculation rigorously enforces the NVE condition or ergodicity. We are interested in achieving a constant temperature and motion characteristics, which should have the same nonlinearity as that shown in the MD simulations. For this reason, we call the thermal condition that we achieved a semi-thermal equilibrium. A study of the ergodicity will be conducted in further work. The parameter set for Eqs. (41)–(42) is given in Table E1 of Supplementary Info. E. Bigger the nodes, more severe memory effect is expected, however, the integration are not no longer essential with heat diffusion damping and its proper parameter set.

To confirm the versatility of our approach, longer SWCNTs are tested with different node lengths. (5,5) SWCNTs with length of 15 nm are calculated using MD simulation with the same simulation condition introduced in Supplementary Info. E, and the results are processed as a simple bead string to create the input data for the CGMD simulation. The given temperature is 300 K, and the same damping algorithm is applied to two different CG particles which are corresponding to 60 and 120 carbon atoms. This approach gives the intended semi-thermal equilibrium condition, as shown in Supplementary Info. E. The results show a constant temperature profile with a longer duration than those obtained in other calculations. The simulations were performed under the perfect rigid fixation condition. With a slightly modified parameter set, the suggested algorithm shows good versatility with SWCNTs with different lengths.

For a simulation with UA60 model, the required simulation time was approximately a maximum of 2 h with a 1.6 GHz Intel Core i5 CPU.

## Discussion

The CG modeling of SWCNTs has been examined to reproduce the nonlinear dynamics. SWCNTs with lengths of 8 nm and 15 nm were modeled with three different CG particle sizes at different temperature conditions in the cantilevered condition. For all of these simulations, the initial conditions are obtained from MD simulations. We found that the characteristics of the dynamics from the conventional CGMD simulation are not consistent with those of the MD simulation due to the random noise from the thermostatting algorithm and the lack of complexity of the potential energy for reproducing the nonlinear bending motion. The effect of the additional thermostat is overwhelming, so the macroscopic motion from free thermal vibration is not preserved. The discrepancy between the simple bead spring model in CGMD and the initial condition from MD simulation is resolved using an additional force suggested from the diffusion of the cross-correlated state of the bond length and angle. The modified equation of motion using heat diffusion of the cross-correlated state maintains its dynamics to be very close to thermal equilibrium, and its macroscopic motion is proved to be consistent with the nonlinear motion observed in the atomic MD simulation. The new method was also shown to be effective for the longer node length with a larger time step than the conventional CGMD simulation including dynamics that are almost perfectly synchronized with the dynamics of the MD simulations. The precise reproduction of the nonlinear motion of SWCNTs in the improved CGMD simulation shows that the causality of nonlinear motion of SWCNTs can be deeply related to the internal heat diffusion in the THz range.

The suggested algorithm described by Eqs. (41)–(42) is clearly different from DPD modeling because it includes random noise and dissipation as a single term. This algorithm is supposed to eliminate the possible evolution of artificial drifts presumed to be due to the cross-correlated state between the different potential energy functions as discussed in previous studies^37,54^ For MD simulations, the drift is assumed to be dissipated as a small amount of perturbation in the THz range so that CG modeling can adapt this constraint to reduce the strong cross-correlation using a diffusion process. Although it is not fully elucidated at the level of phonons and second sound, the almost perfect synchronized motion of the CGMD and MD simulation with the constant temperature profile in Fig. 4 proves that an exact damping or scattering mechanism related to the diffusion can exist. On the other hand, the importance of the optical mode in CG modeling and the macroscopic dynamic characteristics has been mentioned in several papers^24,30,55^, and the memory effect that includes the entropy such as Zwanzig SDE^47,55^ or approximation from the second fluctuation theorem^21^ can provide a more precise expression for calculating the dynamics from the second sound as a further approach. Understanding the role of cross-correlation damping on the reversibility with constant temperature and the formulations in the free energy definition^7,50,51^ in more complicate molecular systems is also required. Seeking a fundamental understanding to obtain precise parameters will enable engineering the nonlinearity at the nanoscale^43,56,57^.

A modified equation of motion with cross-correlation diffusion may lead to several related theories that can provide better a methodology to obtain the parameters in Eqs. (41)–(42). Even though the range of the parameters is conjectured from the cross-correlation in Fig. 2, the process of determining the parameter sets is rather close to the manual method. These parameters depend strongly on various conditions such as the node length, temperature and boundary rigidity. One powerful approach is iterative Boltzmann inversion (IBI), but it is not affordable for the parameters because the population of the state of IBI is derived from the multiplication of the partition functions, which involves averaging the data which makes the characteristics of cross correlated state is averaged out. Some modification with a Bayesian approach for further numerical fitting would be an interesting connection^5^. From the results of this research, a parameter study based on a rigorous theoretical explanation would ensures the further trials which will offer more specific direction for the improved applications of this method not only to nanotubes but also to more complicated CG mapping which is intricated with memory effect.

## Methods

### Derivation of equation of motion with heat diffusion

When it is presumed that the heat bath works for the discrepancy of simultaneous exertion of multiple harmonic potential energy functions which should be independent each other, it is possible to think about the small amount of kinetic energy involved in their balances from the heat bath. In the main text, we note that the cross correlated condition of the momentum can be a sort of heat source which induce the energy diffusion, $\frac{\partial ^2 p_{i}^{'\ell } p_{i}{'\theta }}{\partial x ^2}$. There is no separated value for $p_{i}^{'\ell }$ and $p_{i}{'\theta }$ in the simulation so that each momentum is designated from the angle and bond length difference in a time step, $v_{\theta }=\Delta \theta / \Delta t$ and $v_{\ell }=\Delta \ell /\Delta t$, respectively. Following is the conventional heat diffusion equation:

45 $$\begin{aligned} \frac{\partial \delta u}{\partial t}= & {} D_0 \frac{\partial ^2 \delta u}{\partial x ^2} \end{aligned}$$

46 $$\begin{aligned} \frac{\partial \delta u}{\partial t}= & {} D\frac{\partial ^2}{\partial x^2}\left( v_{\theta }^2+v_{l}^2+2v_{\theta }v_{l} - v_0^2 \right) , \end{aligned}$$

where $D_0$ is diffusion coefficient of the material and *D* is the parameter that compensates the heat and kinetic energy diffusion. $u=u(z,t)$ is the temperature distribution along tube axis *z* and time *t*. $v_0$ is the background temperature of SWCNT which is equivalent to $v_{\theta }^2+v_{l}^2$ as the given kinetic energy when the system has independent Hamiltonian. We can write diffusion equation as followings:47

Because the kinetic energy has additional term $\delta KE$ from $\delta u = 3m/2k_b \delta KE$, so does the equation of motion. It could be controversial because the momentum that we are dealing with is averaged value from the atomic scale simulation. The assumption is that thermal energy compensates macroscopic motion from heat diffusion process by second sound modulation, and the possibility of this assumption has well shown through the dispersion plot in Fig. 3 of the main text, which indicates the second sound modulation has its certain value in the simple beads system.

The modified kinetic energy as including thermal diffusion condition will be:

48 $$\begin{aligned} KE_{tot}= & {} KE_{0}+\delta KE \end{aligned}$$

49 $$\begin{aligned}= & {} \frac{1}{2} \sum _{i} \left( mv_{i}^2+ D' \delta u_{i} \right) , \end{aligned}$$

where *m* is the mass of the particle, *v* is the velocity and *i* is the number of each node. $D'$ is the value of *D* compensated with the inverse of $3m/2k_b$. $KE_{tot}$ and $KE_0$ are the total kinetic energy of the system and original kinetic energy without heat diffusion for cross correlation damping, respectively. The Lagrangian and the equations of the motion with additional kinetic energy is:

50 $$\begin{aligned} \mathcal L= & {} \frac{1}{2} \sum _{i} m\left( v_{i}^2+\delta v_{i}^{'2} \right) - \phi (r, \theta ), \end{aligned}$$

51 $$\begin{aligned} \frac{d}{dt}\left( \frac{\partial \mathcal L}{\partial p_i}\right)= & {} \frac{\partial \mathcal L}{\partial q_i}, \end{aligned}$$

where $\phi$ is potential energy. As mentioned in the main text, $\delta KE$ is in THz range. It is presumed to be as optical mode, so that the term derived from $\delta KE$ is noted with $\left\langle \right\rangle _{op}$:

52 $$\begin{aligned} m\ddot{l}+\alpha \left\langle \frac{\partial ^2 v_{\theta } }{\partial x^2} \right\rangle _{op}= & {} - \frac{\partial \phi }{\partial l}, \end{aligned}$$

53

### MD and CGMD simulation

The appearance of nonlinear motion of SWCNTs in thermal equilibrium depends on the temperature and aspect ratio. Longer SWCNTs have less motion exchange. If the motion exchange is too infrequent, the examination of suggested model will be inefficient. Shorter SWCNTs make the motion too noisy and extremely complex so that the validation will not be easy. Therefore, a proper choice of SWCNTs and temperature for the simulation ensures that the numerical experiments are convenient. Based on these considerations, (5,5) SWCNTs with a length of 8 nm at 300 K are modeled as a simple bead system. In a previous MD simulation study^40^, the trend of motion exchange clearly depends on the rigidity of the fixed end boundary condition. The fixed end with the Lennard-Jones (LJ) potential function is employed to ensure the affordability of the suggested CGMD simulation. These SWCNTs at 300 K provide exemplary nonlinear characteristic dynamics, as shown in Fig. 1.

The MD simulation was performed with the LAMMPS package^58^ with an adaptive intermolecular reactive bond order (AIREBO) potential function^59^, with a time step of 0.5 *fs*. The Langevin thermostat with a damping coefficient of 0.01 ps is attached to the system during the initial 1 ns. The displacement and velocity data of all atoms are captured after 1 *ns* of relaxation. The rigid end fixation is applied at the bottom with the phantom wall condition, which has a length scale $\sigma$ of 0.89 Å  with the LJ potential function. The rigidity of the end fixation, which is determined by the LJ energy scale, is also applied for CGMD simulation. For comparison, the fixation rigidity has been set with 1 and 5 eV.

To define CGMD beads, the displacement and velocity of every 60 atoms are simply averaged as one lumped mass, i.e., a bead. The target SWCNT has 660 atoms so that the system has 11 lumped masses. It is regarded as a unified atom (UA), which is equivalent to a coarse-grained (CG) particle. However, to obtain proper bending motion, one additional unified atom should be attached next to the fixed end of the simple bead model in the CGMD simulation. In this way, the bending angle between the fixed end and its neighbors can be treated. Additionally, simple bead systems with a lumped mass consisting of 20 and 120 atoms are examined. The bond length for the CG particle incorporating 20 carbon atoms (UA20) is 2.42 Å, and the mass is 240 amu. CG particles for 60 and 120 atoms (UA60 and UA120, respectively) are also used in the case of UA20.

The force constants for bond length and angle spring are $k_{sp} = 220\,\hbox {eV}/{\AA }$ and $k_{ang} = 2200$–2800 $\hbox {eV}\,\AA$ from the parameter study^2^ of each spring, which are obtained from the rigorous measurement using an external force at 0 K. The precise value of $k_{ang}$ is determined by the peak location in the frequency domain. The velocity Verlet algorithm is used with a time step of 0.5*fs* for the case of UA20. A time step of 10*fs* is adapted for UA60 and UA120. The total time spans of the simulations are 250*ns* and $5 \mu \hbox {s}$ for UA20 and UA60/UA120, respectively.

### Cross-correlation condition

In Fig. 5, the changes in the strain, $\Delta _t L$ and angle, $\Delta _t \Theta$, in 50 fs of an arbitral node of a string, which are equivalent to $p_i^\ell$ and $p_i^\theta$ with $1/\Delta t$, respectively, are sampled from MD and CGMD simulations and are processed to show cross-correlation in the frequency domain. For MD simulations, the value of strain and angle of beads model are averaged from the atomic structure of SWCNTs for each node. The cross-correlation during 500 ps is used for FFT. Most of the peaks appeared in the THz range for both CGMD and MD simulations. No significant results were obtained in the range below THz. The CGMD simulation results show more distinctive peaks than the MD simulation results. This means that (1) CGMD has the velocity values caused by angle and bond length, which are strongly correlated in a periodic manner due to the absence of an appropriate constraint, and (2) the MD simulation has a cross-correlation that is not active in the bending frequency range but is active in the optical mode range. In the MD simulation, the force caused by $2\varvec{\ell _i} \varvec{\theta _i}$ is close to a small perturbation in the CG description level, and it is eliminated via an internal heat diffusion process through fluctuation-dissipation in the THz range.

## Supplementary information

Supplementary Figure

Supplementary Information A

Supplementary Information B

Supplementary Information C

Supplementary Information D

Supplementary Information E
