# Supplementary material for: Heat diffusion-related damping process in a highly precise coarse-grained model for nonlinear motion of SWCNT
Source: Sci Rep. 2021 Jan 12;11:563. doi: 10.1038/s41598-020-79200-6 (PMC7804176; doi:10.1038/s41598-020-79200-6)
Supplement: Supplementary file 1 — Supplementary Figure [file 41598_2020_79200_MOESM1_ESM.pptx]

## Slide 1
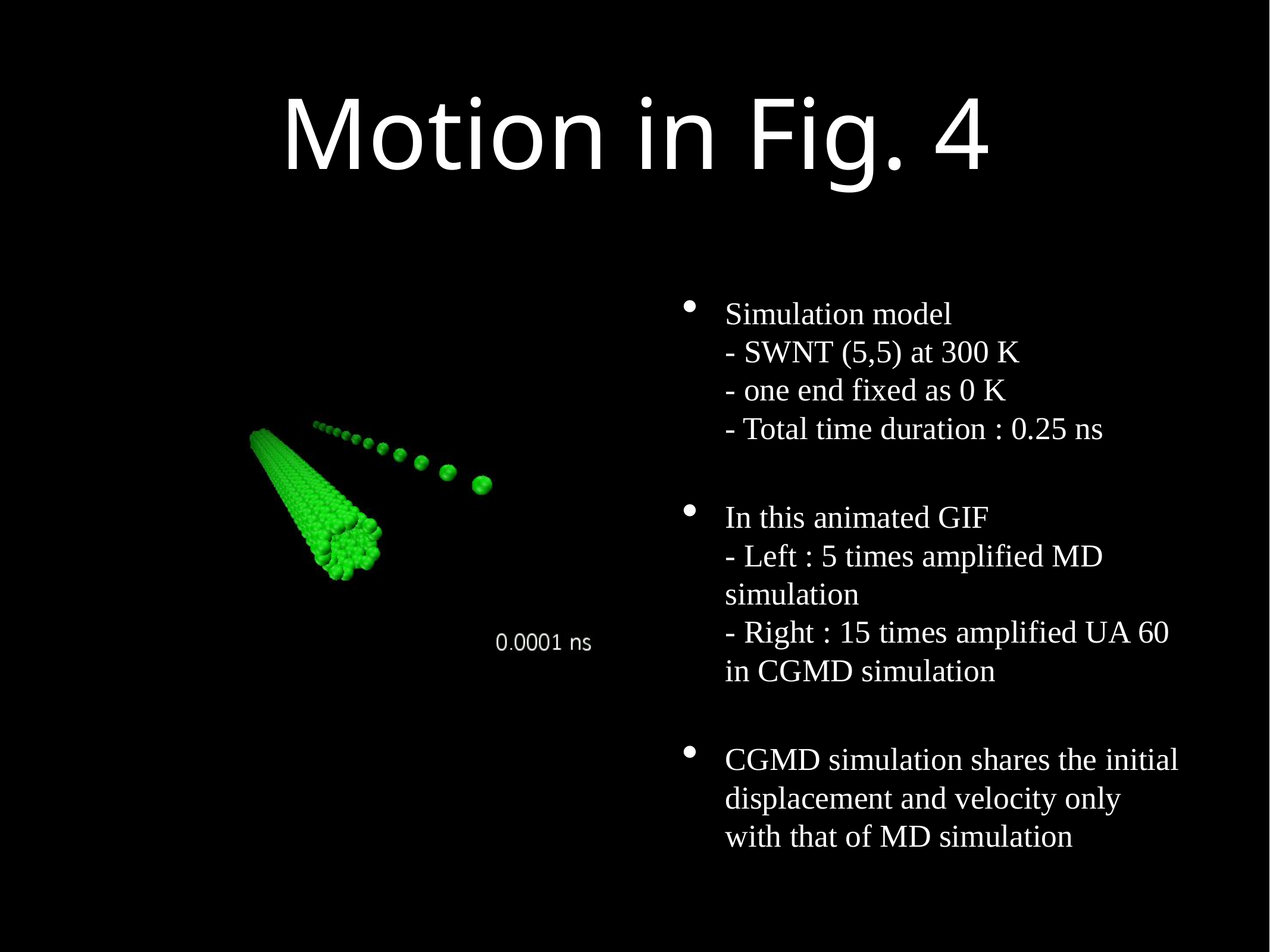

# Motion in Fig. 4
Simulation model
- SWNT (5,5) at 300 K
- one end fixed as 0 K
- Total time duration : 0.25 ns
In this animated GIF
- Left : 5 times amplified MD simulation
- Right : 15 times amplified UA 60 in CGMD simulation
CGMD simulation shares the initial displacement and velocity only with that of MD simulation
