# Supplementary material for: Heat diffusion-related damping process in a highly precise coarse-grained model for nonlinear motion of SWCNT
Source: Sci Rep. 2021 Jan 12;11:563. doi: 10.1038/s41598-020-79200-6 (PMC7804176; doi:10.1038/s41598-020-79200-6)
Supplement: Supplementary file 2 — Supplementary Information A [file 41598_2020_79200_MOESM2_ESM.pdf]

Heat diffusion-related damping process in a highly precise coarse-grained model for nonlinear motion of SWCNT - Supplementary Information A

Heeyuen Koh[1,*], Shohei Chiashi[2], Junichiro Shiomi[2], and Shigeo Maruyama[2,*]

[1]Mechanical and Aerospace Engineering Department, Seoul National University, 1 Gwanak-ro, Gwanak-gu, Seoul, 08826, South Korea
[2]Mechanical Engineering Department, The University of Tokyo, Department of Mechanical Engineering, 7-3-1 Hongo, Bunkyo-ku, Tokyo 113-8656, Japan
*hy_koh@snu.ac.kr
*maruyama@photon.t.u-tokyo.ac.jp

The nonlinearity is the result from the combination of the bending on two perpendicular planes. As the nonlinear bending equation derived from Green Lagrangian strain definition has predicted the free thermal motion of SWCNT in MD simulations,[1] the same Green-Lagrangian strain definition is applied to the simple beads string. Unlike the simple beads string with harmonic potential, the strain definition as a potential energy function gives additional anharmonicity caused by bending on both planes. The definition of the strain energy of i th atom on the surface of SWCNT is:[1,2]

$$U_i = u(z_i, t), \tag{S1}$$

$$V_i = v(z_i, t), \tag{S2}$$

$$W_i = w(z_i, t) + \frac{1}{2}\left(u_{z,i}^2 + v_{z,i}^2\right) - x_i u_{z,i} - y_i v_{z,i}, \tag{S3}$$

here, U_i, V_i and W_i are the displacement along each cartesian coordinate. u,v, w are the displacement of each atom on the SWCNT as the function of the variable z_i. Due to the quasi one-dimensional shape, the displacements along flexural mode in two perpendicular planes are regarded as the function of z_i which is the variable of ith atom in tube length axis. The longitudinal displacement of the tube W_i is also z_i dependent, and it includes the displacement caused by Euler beam assumption $x_i u_{z,i}$ and $y_i v_{z,i}$ so that the governing equation can describe bending motion on two perpendicular planes at the same time. The direct implementation of Eq. (S3) into simple beads system introduces a new variable set with averaged coordinate $\boldsymbol{R_j} = 1/N \sum_i^n \boldsymbol{r_i}$:

$$\boldsymbol{\xi_j} = \boldsymbol{R_j} - \boldsymbol{R}_{j-1}, \tag{S4}$$

$$\boldsymbol{\ell}_i = \left(\xi_{z,i} + 1/2\xi_{x,i}^2 + 1/2\xi_{y,i}^2\right)\boldsymbol{e}_{\ell,i}, \tag{S5}$$

$$\boldsymbol{\theta}_i\left(\boldsymbol{\xi}_i, \boldsymbol{\xi}_{i+1}\right) = \theta_{\boldsymbol{\xi}_i,\boldsymbol{\xi}_{i-1}}\boldsymbol{e}_{\theta,i}, \tag{S6}$$

where,$\boldsymbol{r}_i$ is the vector for the location of i th atom in cartesian coordinate and $\boldsymbol{R}_j$ is that of j th bead in a simple beads system which is averaged from N atoms. N is the number of atoms per a coarse grained particle. Each bead does not have any overlapping for its averaging. The approximation of differentials in Eq. (S3) for bead system can be the difference between neighbour unit atoms, $\boldsymbol{\xi_j}$ as in Eq. (S4), then simple beads system is described by two sets of variables $\boldsymbol{q} = \{\boldsymbol{\ell}_i, \boldsymbol{\theta_i}\}$ as in Eq. (S5) and Eq. (S6). Subscript of $\boldsymbol{\xi_j}$ is its component in each cartesian coordinate. The bond length variable, $\boldsymbol{\ell_i}$ is equivalent to first three terms in Eq. (S3) and angle variable, $\theta_{\boldsymbol{\xi}_i,\boldsymbol{\xi}_{i-1}}$ is same to the rest of terms or just angle between two adjacent vectors $\boldsymbol{\xi}_{j-1}$ and $\boldsymbol{\xi}_j$, as noted as $\theta_{\boldsymbol{\xi}_{i-1},\boldsymbol{\xi}_i}$ which is composed of three beads.

The total energy and potential energy of MD simulation model and Eq. (S3) expressed using CG description is:

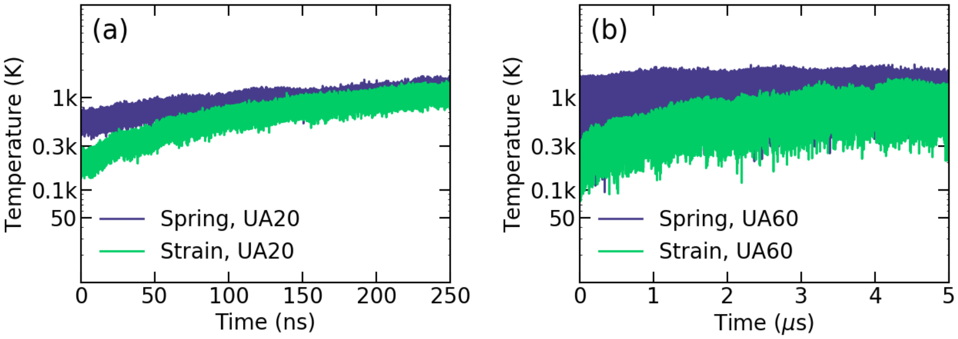

Figure A1. Temperature calculated from spring and strain CGMD simulation without modification and Langevin thermostatting. Blue line is conventional CGMD simulation with harmonic potential function and green is strain CGMD simulation result: (a)UA20, (b)UA60.

$$L = \frac{1}{2}\Sigma_i m\left(\frac{d\boldsymbol{R_i}}{dt}\right)^2 - \Sigma_i U_{i,G.E.} - \Sigma_i U_{i,int}, \tag{S7}$$

$$U_{i,G.E.} = \frac{1}{2}k_\theta(\theta_i + \ell_i)^2, \tag{S8}$$

where, i is node number, m is the same mass for a bead to CGMD modeling. $U_{i,G.E.}$ and $U_{i,int}$ are potential energy defined by Eq. (S3) and internal energy which is not included in CG description, respectively. According to Eq. (S8), the strain definition offers not only the coupling between bending deformation along the both perpendicular plane but also the combinational energy between bond length and angle, $2\boldsymbol{\ell_i\theta_i}$ simultaneously. The nonlinear bending equation as well deals the coupled state between bond length and angle deformation simultaneously during its derivation process.

The Lagrangian for ideal CG modeling with the deformation defined by Eq. (S5) $\sim$ Eq. (S6) is:

$$L = \frac{1}{2}\Sigma_i m\left(\frac{d\boldsymbol{\ell}_i}{dt}\right)^2 + \frac{1}{2}\Sigma_i I\left(\frac{d\boldsymbol{\theta}_i}{dt}\right)^2 - \Sigma_i U_i^\ell - \Sigma_i U_i^\theta, \tag{S9}$$

where, k_θ and k_ℓ are the spring constants for each deformation $\boldsymbol{\ell}_i$ and $\boldsymbol{\theta}_i$. Potential energies are respectively, $U_i^\theta = 1/2k_\theta\theta_i^2$ and $U_i^\ell = 1/2k_\ell\ell_i^2$. Two independent equations of motion are derived from:

$$\frac{d}{dt}\frac{\partial L}{\partial q_i} - \frac{\partial L}{\partial q_i} = 0. \tag{S10}$$

Please be aware that the CGMD algorithm can not separate the kinetic energy as given in Eq. (S9) so that the equation of motion in Eq. (S10) is not affordable in conventional CGMD simulation. The results of missing the momentum separation using Eq. (S4) $\sim$ (S6) with initial data by MD simulation give the temperature rise profile as shown in Fig. A1, labeled 'strainCGMD'. The harmonic potential energy model from Eq. (S9) with simple length definition instead of strain in Eq. (S3) is named 'springCGMD'. The motion characteristics of CGMD simulation using strain energy has shown the similar motion exchange longer than simple beads system, but both of them are not simply comparable to the MD simulation result because thermal equilibrium is not achieved. However, the additional thermostat is not an answer due to its random force which ruins nonlinear motion trend.

References

1. Koh, H. *et al.* Thermally induced nonlinear vibration of single-walled carbon nanotubes. *Phys. Rev. B* **92**, 024306, DOI: 10.1103/PhysRevB.92.024306 (2015).

2. Ho, C.-H., Scott, R. & Elsley, J. Non planar nonlinear oscillations of a beam : II Free motions. *J. Sound Vib.* **47**, 333–339 (1976).
