# Supplementary material for: Heat diffusion-related damping process in a highly precise coarse-grained model for nonlinear motion of SWCNT
Source: Sci Rep. 2021 Jan 12;11:563. doi: 10.1038/s41598-020-79200-6 (PMC7804176; doi:10.1038/s41598-020-79200-6)
Supplement: Supplementary file 3 — Supplementary Information B [file 41598_2020_79200_MOESM3_ESM.pdf]

Heat diffusion-related damping process in a highly precise coarse-grained model for nonlinear motion of SWCNT - Supplementary Information B

Heeyuen Koh[1,*], **Shohei Chiashi** [2], **Junichiro Shiomi**[2], **and Shigeo Maruyama**[2,*]

[1]Mechanical and Aerospace Engineering Department, Seoul National University, 1 Gwanak-ro, Gwanak-gu, Seoul, 08826, South Korea
[2]Mechanical Engineering Department, The University of Tokyo, Department of Mechanical Engineering, 7-3-1 Hongo, Bunkyo-ku, Tokyo 113-8656, Japan
[*]hy_koh@snu.ac.kr
[*]maruyama@photon.t.u-tokyo.ac.jp

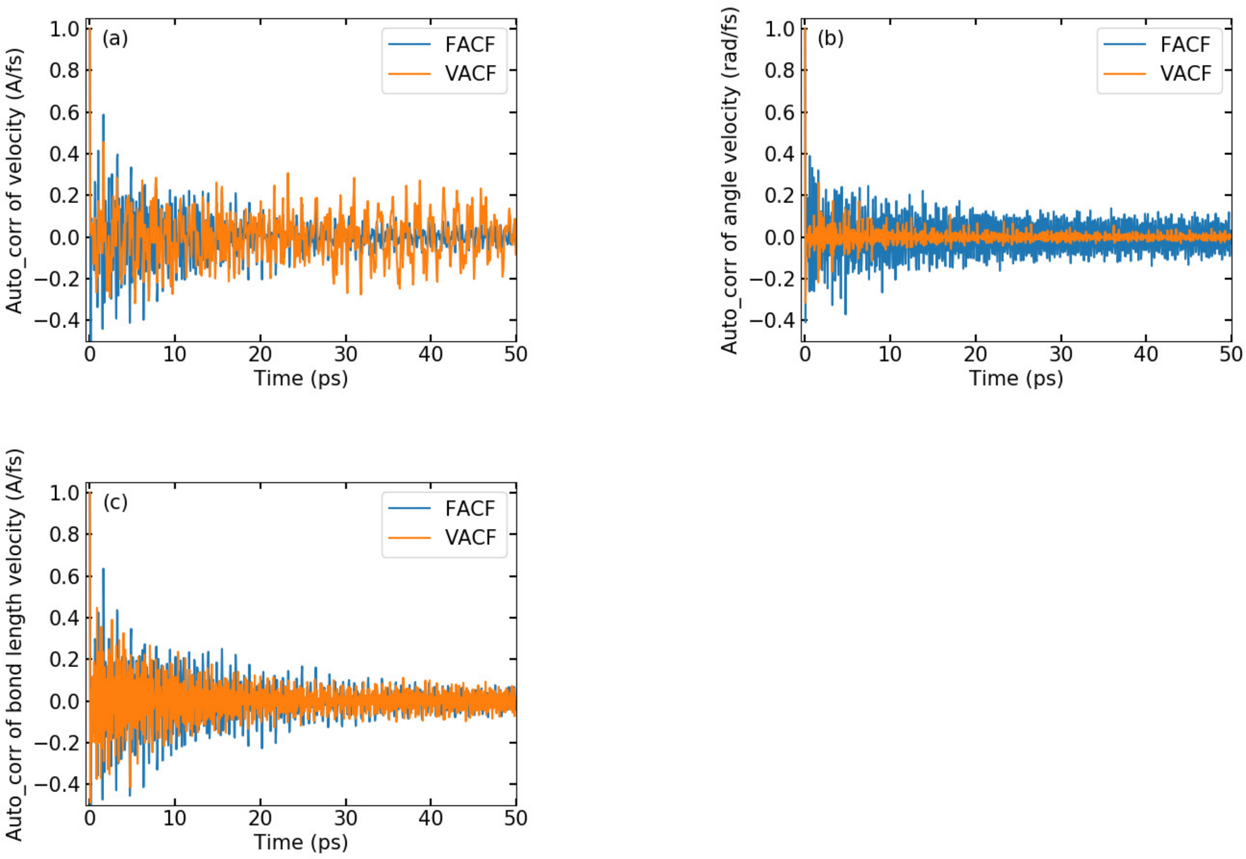

Figure B2. Since the system is dealing with H_{tot} with H_θ and H_ℓ, 3 types of auto correlation for each system are considered: (a) FACF and VACF for H_θ, (b) FACF and VACF for H_ℓ, (c) FACF and VACF for H_{tot}

Above graphs are auto correlations for each type of velocity. All of them show apparently converged to 0, but this trend is

not close to Markovian characteristics, clearer convergence of force should be shown in the auto correlation of bond length and angle velocity. To calculate auto-correlation, the velocity and force of 7th node of simple beads system from MD simulation is collected with time interval 50 fs. The simulation condition of MD simulation is identical as written in the Method section.
