# Supplementary material for: Heat diffusion-related damping process in a highly precise coarse-grained model for nonlinear motion of SWCNT
Source: Sci Rep. 2021 Jan 12;11:563. doi: 10.1038/s41598-020-79200-6 (PMC7804176; doi:10.1038/s41598-020-79200-6)
Supplement: Supplementary file 4 — Supplementary Information C [file 41598_2020_79200_MOESM4_ESM.pdf]

Heat diffusion-related damping process in a highly precise coarse-grained model for nonlinear motion of SWCNT- Supplementary Information C

Heeyuen Koh[1,*], Shohei Chiashi[2], Junichiro Shiomi[2], and Shigeo Maruyama[2,*]

[1]Mechanical and Aerospace Engineering Department, Seoul National University, 1 Gwanak-ro, Gwanak-gu, Seoul, 08826, South Korea
[2]Mechanical Engineering Department, The University of Tokyo, Department of Mechanical Engineering, 7-3-1 Hongo, Bunkyo-ku, Tokyo 113-8656, Japan
[*]hy_koh@snu.ac.kr
[*]maruyama@photon.t.u-tokyo.ac.jp

As mentioned in the main text, the velocity of two Hamiltonian system with sharing a momentum makes the definition of each type of momentum to be separated the total velocity using the displace information at each moment. In computer simulation, the momenta can be measured and separated as follows:

$$v_i = (v_\theta + v_l)\,\hat{e}_{v_i}, \tag{S1}$$

$$\|\dot{\theta}\bar{e}_{\dot{\theta}_i}\| = \frac{\Delta_t \theta_i}{\Delta t} \cong \gamma_\theta v_\theta, \tag{S2}$$

$$\|\dot{\ell}\bar{e}_{\dot{\ell}_i}\| = \frac{\Delta_t \ell_i}{\Delta t} \cong \gamma_\ell v_\theta, \tag{S3}$$

here, v_i is the velocity of ith unit mass in CGMD simulation. v_θ and v_l are the velocity caused by angle and bond length. They are the scalar components of total velocity. They would share the unit vector, $\hat{e}_v$ which is the direction of total velocity, v_i. $\dot{\theta}\bar{e}_{\dot{\theta}_i}$ and $\dot{\ell}\bar{e}_{\dot{\ell}_i}$ are, on the contrary, the momenta which are independent each other so that these definition fit to the condition for independent hamiltonian system. Instead of separate those from Eq. (S1), they are directly measured from the simulation by the definitions in the Eq. (S2) and Eq.(S3). $\Delta_t\theta_i$ and $\Delta_t\ell_i$ are the change of angle and bond length in certain time step, Δt which is the inverse of sampling frequency. The amount of the correlation bewteen the independent momenta to contribution of the total velocity is designated as the constant number, γ. This might not be the constant during the whole simulation time, and this variable in function of frequency domain would represent the related condition to memory effect, but rest of explanation is remained for another research with more rigorous theoretical work.

When it is presumed that the heat bath works for the discrepancy of simultaneous exertion of multiple harmonic potential energy functions which should be independent each other, it is possible to think about the small amount of kinetic energy involved in their balances from the heat bath. If the heat has induced by any sort of heat source, it has to be diffused. Following is the conventional heat diffusion equation:

$$\frac{\partial u}{\partial t} = \alpha \frac{\partial^2 u}{\partial x^2}, \tag{S4}$$

where u is the temperature difference ΔT. The heat diffusion equation above can be expressed as kinetic energy format using $k_b T = 3m/2 < v_i^2 >$.

$$\frac{\partial \Delta T}{\partial t} = \frac{3m}{2k_b}\frac{\partial^2}{\partial x^2}\left(v_\theta^2 + v_l^2 + 2v_\theta v_l - v_0^2\right), \tag{S5}$$

where v_0 is equivalent to $v_\theta^2 + v_l^2$ as given kinetic energy of the system when it keeps the independency as Hamiltonian system. We can rewrite the Eq. (S5) as followings:

$$\delta T = \alpha \int \frac{\partial^2 T}{\partial x^2} dt = \alpha \int \frac{\partial^2 v_\theta \cdot v_l}{\partial x^2} dt. \tag{S6}$$

Because the kinetic energy has additional term, so does the equation of motion. The kinetic energy modified by diffusion of cross correlation $2v_\theta v_l$ is designated with δ as δT so that the fraction of the kinetic energy modified from the heat diffusion can be noted as $\delta T = \frac{3m}{2k_b} \delta KE$. It could be controversial because the momentum that we are dealing with is averaged value from atomic simulation to consider coarse grained system. The assumption is that thermal energy compensation from heat diffusion must occupy small amount of kinetic energy of real system without disturbing coarse graining, and the possibility of this assumption has well shown through the dispersion plot of $\frac{\partial^2 v_\theta \cdot v_l}{\partial x^2}$ in Fig. 3 of main text, which indicate the second wave has its certain value even in the simple beads system.

The modified kinetic energy as including thermal diffusion condition will be:

$$KE_{tot} = KE_0 + \delta KE \tag{S7}$$

$$= \frac{1}{2} \sum_i \left(m v_i^2 + m \delta v_i'^2 \right). \tag{S8}$$

The Lagrangian with additional kinetic energy is:

$$\mathscr{L} = \frac{1}{2} \sum_i m \left(v_i^2 + \delta v_i'^2 \right) - \phi(r, \theta), \tag{S9}$$

$$\frac{d}{dt} \left(\frac{\partial \mathscr{L}}{\partial p_i} \right) = \frac{\partial \mathscr{L}}{\partial q_i}. \tag{S10}$$

The variable for Lagrangian are $p_i = I\dot{\theta}$ or $m\dot{l}$, and $q_i = \theta$ or l. Lets remind the velocity v_θ and v_l belongs to each node i just like the Eq. (S1) does. We put the parameter for this proportionality as γ in Eq. (S1). Then the equation of motion become like followings:

$$\frac{d}{dt} \left(\frac{\partial \delta v_i'^2}{\partial \dot{\theta}} \right) = \gamma \frac{d}{dt} \frac{\partial^2 \delta v_l dt}{\partial x^2}, \tag{S11}$$

$$\frac{d}{dt} \left(\frac{\partial \delta v_i'^2}{\partial \dot{l}} \right) = \gamma \frac{d}{dt} \frac{\partial^2 \delta v_\theta dt}{\partial x^2}. \tag{S12}$$

here, δv_l and δv_θ is the result of the time integration from the heat diffusion equation as shown in Eq. (S8) so that these two terms are expressed as its results of integration $\Delta_t l$ and $\Delta_t \theta$, respectively. The rest of terms are disappeared because $\frac{\partial^2 v_\theta}{\partial x^2}$, for example, become constant after processing $\frac{\partial}{\partial \dot{\theta}}$. Therefore, equation of motion becomes as followings:

$$m\ddot{l} + \alpha \frac{\partial^2 \Delta_t \theta}{\partial x^2} = -\frac{\partial \phi}{\partial l}, \tag{S13}$$

$$I\ddot{\theta} + \alpha' \frac{\partial^2 \Delta_t l}{\partial x^2} = -\frac{\partial \phi}{\partial \theta}. \tag{S14}$$

They are equivalent to the algorithm that has suggested in the manuscript with the assumption that the additional terms should be remained in THz range. The parameters for algorithm must include heat diffusion coefficient and the compensation between $v_{\theta,l}$ to $\dot{\theta}$ or $\dot{l}$.
