# Supplementary material for: Heat diffusion-related damping process in a highly precise coarse-grained model for nonlinear motion of SWCNT
Source: Sci Rep. 2021 Jan 12;11:563. doi: 10.1038/s41598-020-79200-6 (PMC7804176; doi:10.1038/s41598-020-79200-6)
Supplement: Supplementary file 5 — Supplementary Information D [file 41598_2020_79200_MOESM5_ESM.pdf]

Heat diffusion-related damping process in a highly precise coarse-grained model for nonlinear motion of SWCNT- Supplementary Information D

Heeyuen Koh[1,*], Shohei Chiashi [2], Junichiro Shiomi[2], and Shigeo Maruyama[2,*]

[1]Mechanical and Aerospace Engineering Department, Seoul National University, 1 Gwanak-ro, Gwanak-gu, Seoul, 08826, South Korea
[2]Mechanical Engineering Department, The University of Tokyo, Department of Mechanical Engineering, 7-3-1 Hongo, Bunkyo-ku, Tokyo 113-8656, Japan
[*]hy_koh@snu.ac.kr
[*]maruyama@photon.t.u-tokyo.ac.jp

1 Animation with CG model and MD simulation results

Attached pptx file has the information of the simulation condition with animated gif.

2 Number of rotation in long duration

The motion characteristics are compared with the two evaluation tests 1) the trajectory of the motion during initial 25 ns, and 2) the rate of motion exchange for 0.2 μs. Both conditions can be directly compared to that from MD simulation and theoretical approximation. The rotation profiles using angular velocity of the tip of the tube during initial 25 ns are presented in Fig. D1 with the result from MD simulation. The angular velocity whose value is more than 5 rad/ps means the motion is in planar bending, the case with the value less than that indicates the tube in whirling motion. It has positive value in clockwise rotation, and negative value when it is rotating in the counter clockwise. The result with LJ potential fixation has more close motion exchange to the MD simulation as shown in Fig. D1 (d). In case of Fig. D1 (b) and (c), the result at 300 K with rigid boundary condition and LJ potential fixation with ε at 5 eV has less rotation exchange compared to the Fig. D1 (d) whose boundary condition is with of 1 eV. The motion characteristics is clearly dependent on the rigidity of the fixation like the result in MD simulation.

The rotation profile during initial 25 ns does not guarantee the nonlinear feature of the motion in longer time period so that the number of rotation exchange during 250 ns is observed and shown in Fig. S3. In MD simulation and the theoretical approach whose result is fitted to MD simulation[1] suggest that the total number of rotation exchange during 250 ns should be around 10 times. In Fig. D2, the rotation exchange number with ε = 1 eV is approximately more than 10 times. And fixation with = 5 eV and perfect rigid boundary show almost no rotation exchange. We can conclude that there is no change of the trends in motion characteristics so that the firstly given dynamics during initial 25 ns are maintained as well during 250 ns. The nonlinear motion characteristics are firmly stable with constant temperature and total energy during the whole simulation time in all CGMD simulation.

References

1. Koh, H. *et al.* Thermally induced nonlinear vibration of single-walled carbon nanotubes. *Phys. Rev. B* **92**, 024306, DOI: 10.1103/PhysRevB.92.024306 (2015).

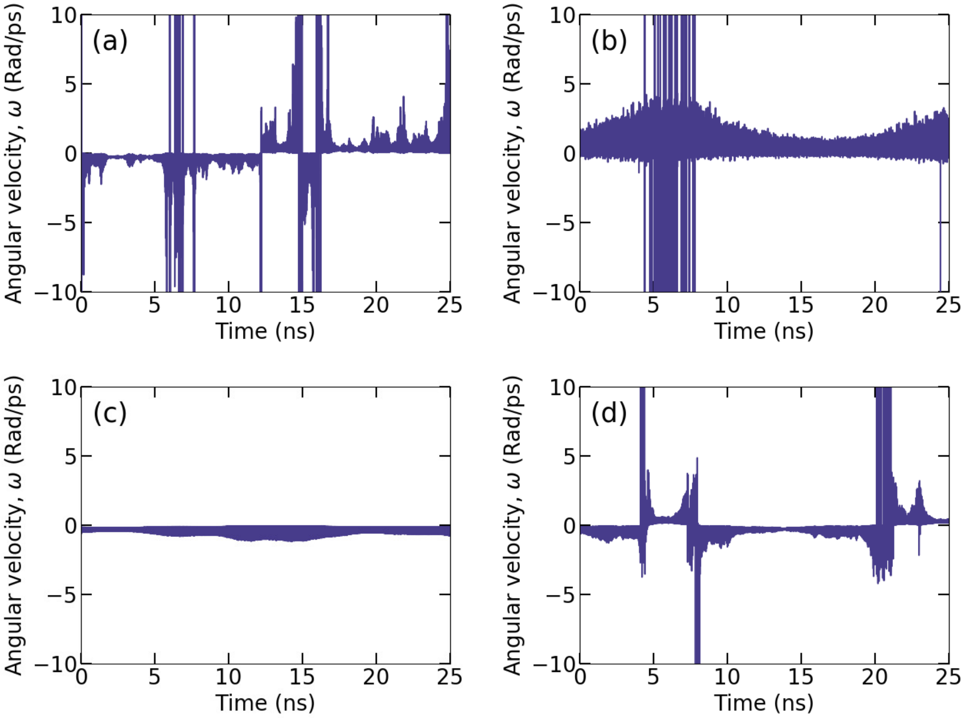

Figure D1. Angular velocity during 25 ns: (a) MD simulation at 300 K, (b) Strain CGMD with UA 60 at 300 K, (c) Strain CGMD with UA 60 and LJ potential boundary with ε = 5 eV at 300 K, (d) Strain CGMD with UA 60 and LJ potential boundary with ε = 1 eV at 300 K.

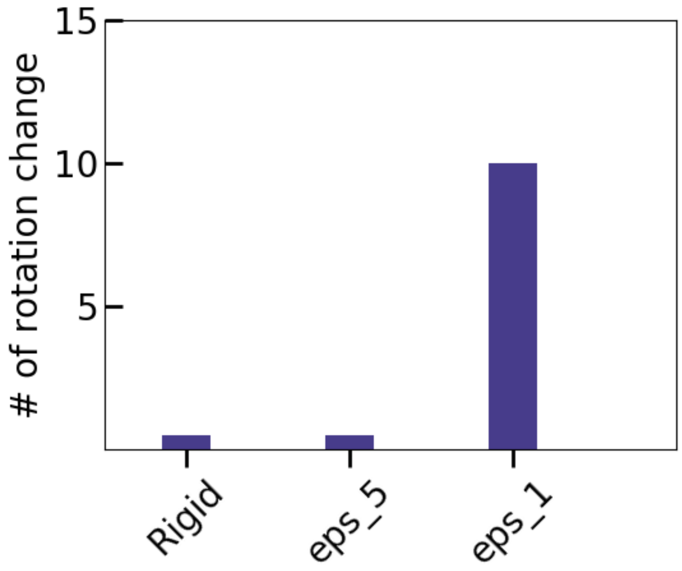

Figure D2. The count number how many time the motion has changed during 25 ns. CGMD model with 1 eV fixation has better similarity to MD simulation.
