# Supplementary material for: Heat diffusion-related damping process in a highly precise coarse-grained model for nonlinear motion of SWCNT
Source: Sci Rep. 2021 Jan 12;11:563. doi: 10.1038/s41598-020-79200-6 (PMC7804176; doi:10.1038/s41598-020-79200-6)
Supplement: Supplementary file 6 — Supplementary Information E [file 41598_2020_79200_MOESM6_ESM.pdf]

Heat diffusion-related damping process in a highly precise coarse-grained model for nonlinear motion of SWCNT - Supplementary Information E

Heeyuen Koh[1,*], Shohei Chiashi[2], Junichiro Shiomi[2], and Shigeo Maruyama[2,*]

[1]Mechanical and Aerospace Engineering Department, Seoul National University, 1 Gwanak-ro, Gwanak-gu, Seoul, 08826, South Korea
[2]Mechanical Engineering Department, The University of Tokyo, Department of Mechanical Engineering, 7-3-1 Hongo, Bunkyo-ku, Tokyo 113-8656, Japan
[*]hy_koh@snu.ac.kr
[*]maruyama@photon.t.u-tokyo.ac.jp

1 Limits of cross correlation damping with longer node length and parameter table

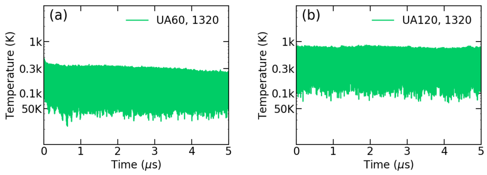

Figure E1. Temperature profile of CGMD for (5,5) 16 nm long SWCNT at 300 K: (a) UA60, (b)UA120.

The upper limit of the node length which is examined by (5,5) 16 nm SWCNT is 14.52 Ang. The deviation of the temperature is too wide, there is clear boundary how much node length can be expanded as well as the time step size whose maximum value is 0.01 fs. The parameters for each node length is followed in next page.

2 Parameter set for each simulation

Table E1. The value of parameter α and α'

CG model	Length (nm)	Temp. (K)	Rigidity (eV)	α (eV/Å)	α' (rad)
UA20	8	50	0 K	19.68	96.52
		100		9.84	48.26
		300		9.84	48.26
UA60	8	100	0 K	1.44	7.24
		300	0 K	1.31	2.14
		300	1	0.42	1.67
		300	5	0.63	2.51
	15	300	0 K	3.93	6.43
UA120	15	300	0 K	1.31	1.07
